# Reversible Charge-Mediated Capture and Release of Extracellular Vesicles Using Giant Unilamellar Vesicles

**DOI:** 10.1021/acsomega.6c00387

**Published:** 2026-08-13

**Authors:** Marinella Pinelli, Gabriele Schiavi, Dalia Tarantino, Luca Valer, Martina Cortese, Michela Notarangelo, Miriam Romano, Annalisa Radeghieri, Lucia Paolini, Paolo Bergese, Vito G. D’Agostino, Martin M. Hanczyc, Silvia Holler

**Affiliations:** † Cellular Computational and Integrative Biology Department, CIBIO, 19034 University of Trento, Via Sommarive 9, Povo, 38123, Italy; ‡ Department of Molecular and Translational Medicine, 9297 University of Brescia, Brescia 25123, Italy; § Center for Colloid and Surface Science (CSGI), Sesto Fiorentino (FI) 50019, Italy; ∥ Department of Medical and Surgical Specialties, Radiological Sciences and Public Health, 9297 University of Brescia, Brescia 25123, Italy; ⊥ Chemical and Biological Engineering, University of New Mexico, Albuquerque, New Mexico 87106, United States

## Abstract

Extracellular vesicles (EVs) play crucial roles in intercellular communication and represent promising therapeutic vehicles, yet current isolation and manipulation methods often irreversibly alter their properties. This study presents a charge-mediated approach for the reversible interaction and release of EVs using Giant Unilamellar Vesicles (GUVs) exploiting tailored surface charges. Negatively charged EVs can be selectively bound through electrostatic interactions with positively charged GUVs, forming stable aggregates. Critically, these interactions are reversible upon the introduction of a negatively charged moiety, oleic acid, which disaggregates the complexes and releases the vesicles. Selective fusion events between oppositely charged GUVs allow modulation of internal composition, while GUVs–EVs interactions remain nonfusogenic. This reversible, charge-based platform offers advantages over current EVs’ capture methods, including simplicity, low cost, and the potential for a separation method without permanent labeling.

## Graphical abstract

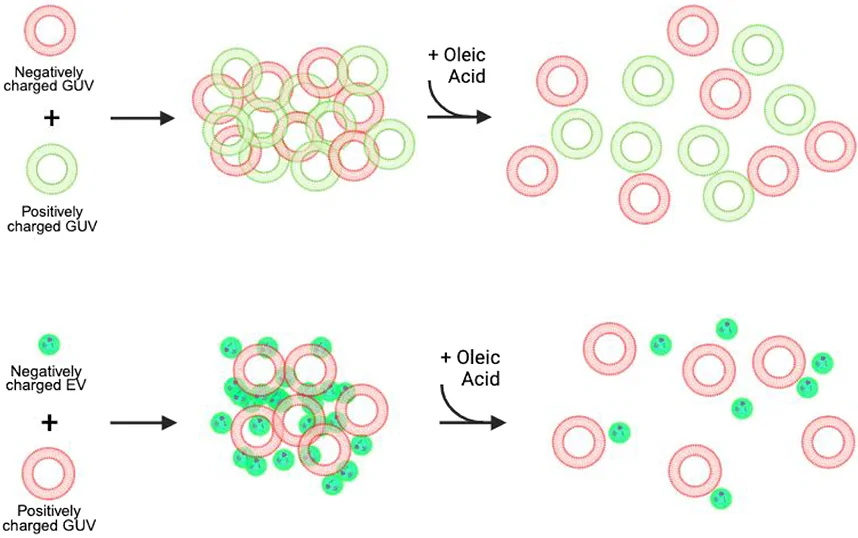

## Introduction

Extracellular vesicles (EVs) are nanoscale vesicles secreted by virtually all cell types,^1^ playing essential roles in intercellular communication^2^ by transporting bioactive molecules such as proteins, lipids, and nucleic acids.^3–5^ EVs are highly heterogeneous in size, typically ranging from 30 nm to over 1000 nm,^6^ and are commonly categorized into subtypes^7^ such as exosomes, microvesicles, and apoptotic bodies based on their biogenesis and dimensions.

Due to their native origin and inherent biocompatibility,^8^ EVs have attracted significant interest for use as disease biomarkers^9^ and in therapeutic delivery systems.^10,11^ However, their clinical and research applications are currently limited by challenges in isolation,^12^ characterization,^7^ and functional modification.^13^ A wide range of protocols have been developed for the isolation of EVs, each aiming to balance purity, yield, and practicality. These methods, including ultracentrifugation,^14^ size-exclusion chromatography,^15^ and immunoaffinity capture,^16^ often suffer from high operational or equipment costs, labor-intensive procedures, low yield, or potential alteration of the EVs’ integrity.^13,17,18^ In particular, possible alterations to EVs’ native structure represent a significant challenge for applications that require the preservation of EVs’ natural properties, such as therapeutic delivery or biomarker discovery.

EVs exhibit a similar surface charge,^19^ typically negative due to the presence of phosphatidylserine^20^ and other anionic lipids^21^ in their membranes. Previous research has exploited electrostatic interactions to isolate EVs from cell culture media and plasma, filtering out impurities, though potentially compromising their membrane integrity.^22–25^ For this study, we chose to take advantage of the negative charge of EVs to promote their interaction with artificial vesicles, which offer a tunable and modular platform for experimental manipulation. The chosen artificial vesicles were Giant Unilamellar Vesicles (GUVs). GUVs are artificial giant lipid vesicles, typically ranging from 1 to 100 μm in diameter, that have long served as cell^26^ and biomimetic membrane models.^27,28^ Their size allows for direct visualization and manipulation using microscopy, and their lipid composition can be precisely controlled^29^ to mimic various biological membrane environments and to create innovative technologies.^30^

In this work, GUVs were engineered to interact with EVs and form aggregates via charge-mediated mechanisms. This approach was chosen for its simplicity and, as demonstrated experimentally, its reversibility. Specifically, the addition of negatively charged oleic acid to the EVs–GUVs complexes enabled the release of EVs without any evidence of compromised structural integrity.

## Results

To investigate the role of surface charge in the interaction between GUVs and EVs, we first prepared a test system and analyzed the interaction of GUVs with either similar or opposite surface charges. Two distinct populations of GUVs with well-defined positive or negative surface charges were generated. Negatively charged GUVs were generated by incorporating 1-palmitoyl-2-oleoyl-*sn*-glycero-3-phospho-l-serine (POPS) into their lipid mixture, mimicking a surface charge profile predicted as “noninteracting” with negatively charged membranes. Positively charged GUVs were instead formed by adding 1,2-dioleoyl-3-trimethylammonium-propane (DOTAP), creating a membrane environment favorable for electrostatic interaction with the negatively charged membranes.

l-α-Phosphatidylethanolamine-N-(7-nitro-2-1,3-benzoxadiazol-4-yl) (NBD-PE), which allows to visualize the GUV membrane as green, and 1,2-dioleoyl-*sn*-glycero-3-phosphoethanolamine-N-(Cyanine 5) (Cy5-PE), which allows to visualize the GUV membrane as red, were used as fluorescent lipid markers to distinguish between different populations. Following their preparation, equal volumes of negatively and positively charged GUVs were mixed to allow potential aggregation driven by electrostatic interactions. The formation of aggregates was assessed by confocal fluorescence microscopy and quantitatively analyzed using ImageStream flow cytometry (see Figure 1).

**Figure 1:**
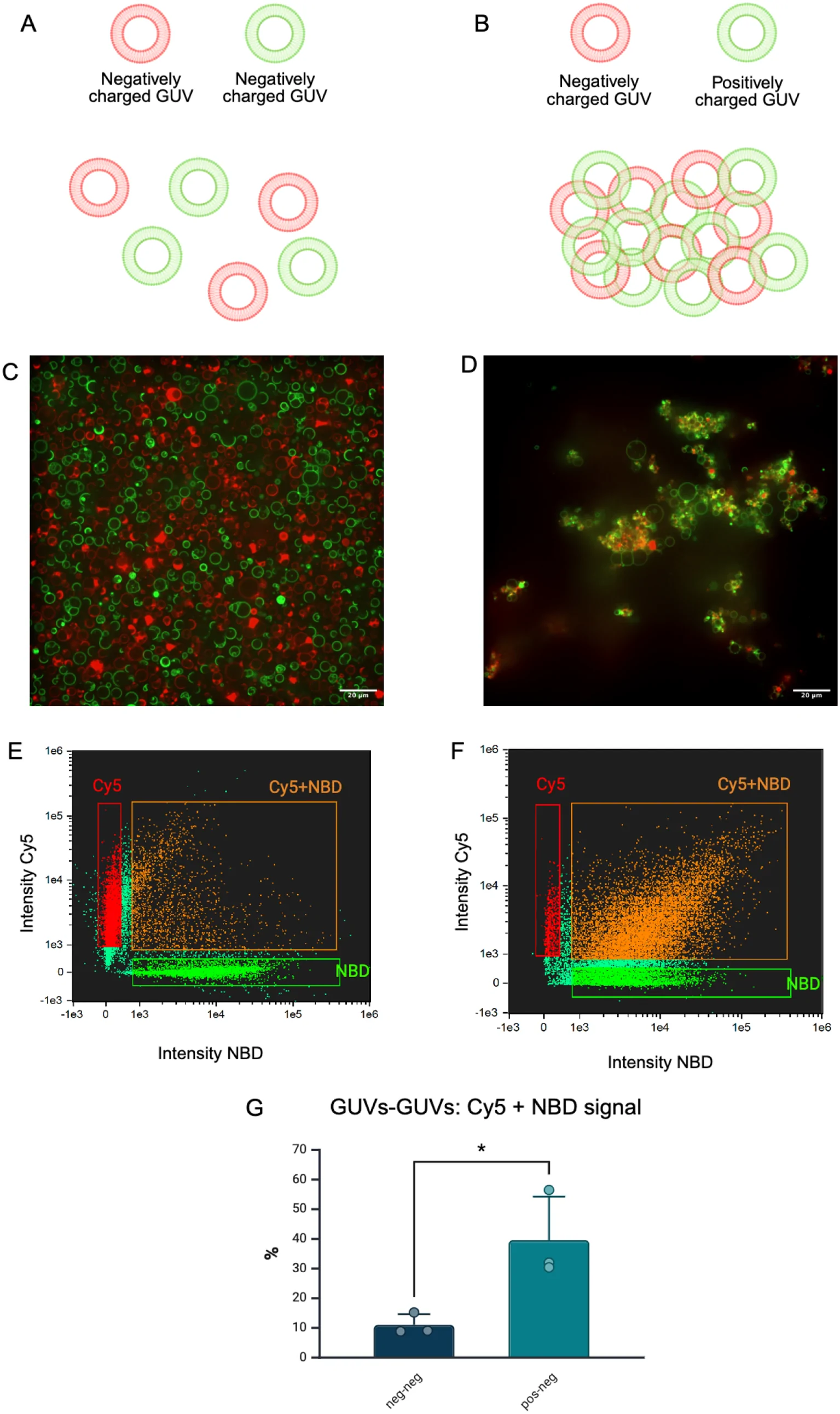
Interaction of charged GUVs. Schematic representation of the interaction between similarly charged GUVs (A) and oppositely charged GUVs (B). Confocal microscopy images of GUV mixes: no interaction of GUVs with the same charge (C) and interaction of GUVs with opposite charges (D). (E) and (F): Flow cytometry fluorescence comparison plots of (C) and (D) samples, respectively. (G) Bar graph representing the percentages of objects showing signals in both the far-red (Cy5) and green (NBD) channels, obtained by flow cytometry analysis for negative–negative (neg–neg) and negative–positive (neg–pos) GUV mixes. Each experiment was repeated at least 3 times; error bars represent the standard deviation; *p* > 0.05 ns, *p* < 0.05*, *p* < 0.01**, *p* < 0.001***. Scale bar: 20 μm.

As shown in the confocal microscopy images in Figure 1, the mixing of oppositely charged GUVs, DOTAP-enriched positively charged and POPS-enriched negatively charged, led to the formation of aggregates (having a mean diameter of around 20–25 μm), accompanied by a marked increase in colocalization of green and red vesicles (Figure 1D). In contrast, the mixing of two negatively charged GUV populations (Figure 1C) resulted in minimal interaction, consistent with electrostatic repulsion. These findings demonstrate that surface charge plays a pivotal role in regulating GUV–GUV interactions. Especially in the negative–negative samples, a certain degree of phase separation between DPPC and DOPE lipids was observed, as evidenced by the lipid dye polarization. However, since this did not affect aggregate formation, we continued using this lipid mixture due to its superior stability compared to other formulations used for GUV formation.

The microscopy results were further validated through flow cytometric analysis using ImageStream. In the fluorescence intensity plots, it is evident that when two negatively charged populations are mixed, they form two distinct, nonoverlapping distributions, indicating minimal to no interaction between the vesicles (Figure 1E). In contrast, when populations with opposite charges are combined, a third distribution emerges, characterized by objects exhibiting double fluorescence signals (Figure 1F). The statistical increase in the double-positive signal in the case of mixed positive–negative populations (from 11 ± 4% to 40 ± 15%) was confirmed to be significant (see Figure 1G).

Following the investigation of GUV aggregation, we applied the same methodology to analyze the charge-mediated binding of EVs. EVs were isolated from the GFP-expressing U87 MG cell line so that their internal lumen was fluorescent in the green channel. Negatively and positively charged GUVs were prepared and subsequently incubated with purified EVs. The resulting mixtures were imaged and analyzed by using confocal microscopy and ImageStream flow cytometry.

As shown in Figure 2, prior to mixing, extracellular vesicles (EVs) were quantified and their size distribution was characterized using Nanoparticle Tracking Analysis (NTA) (Figure 2C). When negatively charged giant unilamellar vesicles (GUVs) were mixed with EVs, no aggregation was observed (Figure 2D). In contrast, the same amount of positively charged GUVs readily aggregated upon mixing with EVs (Figure 2E). This observation was further supported by ImageStream analysis, which confirmed that double-fluorescent aggregates formed exclusively when oppositely charged populations, of positive GUVs and EVs, interacted (Figure 2F and G). Quantitative data from ImageStream also demonstrated a statistically significant difference between the absence of aggregates in the similarly charged GUVs–EVs system and the pronounced aggregation in the oppositely charged GUVs–EVs systems, with an increase in double-positive signal from 8.3 ± 1.7% to 62.3 ± 19.2% (Figure 2H).

**Figure 2:**
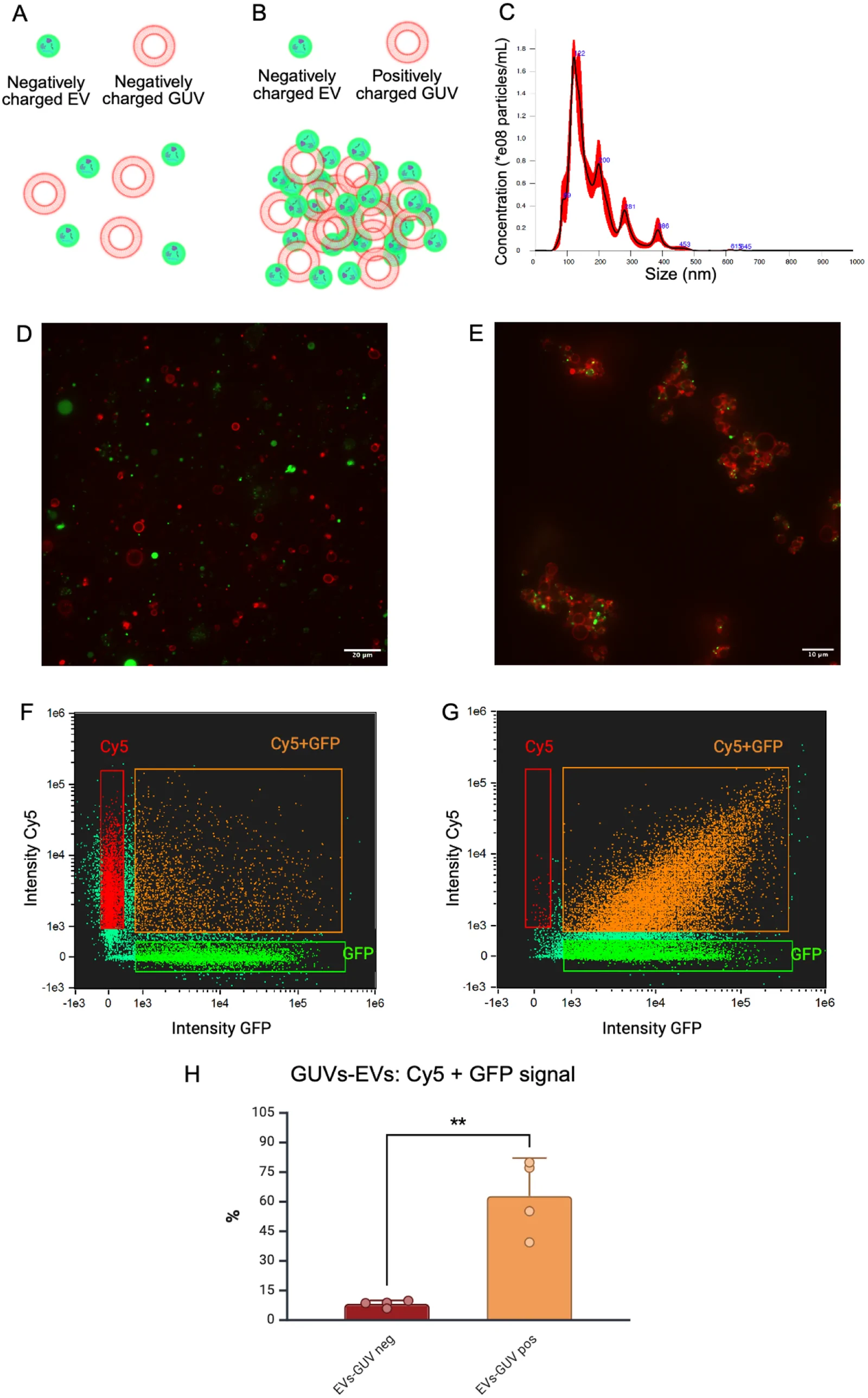
Interaction of charged GUVs with EVs. Schematic representation of the interaction between negatively charged GUVs and EVs (A) and positively charged GUVs with EVs (B). EV size and concentration (C) were assessed by NTA (Nanoparticle Tracking Analysis). Confocal microscopy images of GUVs–EVs mixes represented in (A) and (B): no interaction of GUVs–EVs with similar charges (D) and interaction of GUVs–EVs with opposite charges (E). (F) and (G): flow cytometry fluorescence comparison plots of (D) and (E) samples, respectively. (H) Bar graph showing the percentages of objects showing signals in both the far-red (Cy5) and green (GFP) channels obtained by flow cytometry analysis for EVs–GUV negative mixes and EVs–GUV positive mixes. Each experiment was repeated at least 3 times; error bars represent the standard deviation; *p* > 0.05 ns, *p* < 0.05*, *p* < 0.01**, *p* < 0.001***. Scale bars: 20 μm (D) and 10 μm (E).

Based on the observed charge-mediated interactions between GUV–GUV and GUV–EV, the reversibility of these electrostatic interactions was subsequently investigated. The GUV–GUV aggregates were incubated with ionic entities predicted to interact with their surface charge: oleic acid, sodium dodecyl sulfate (SDS), and didodecyldimethylammonium bromide (DDAB). Detergents can be used not only to solubilize vesicles’ membranes but also to interact with and modify their surface charge.^31,32^ Since oleic acid and SDS carry a negative charge at pH 7.2, they were expected to interact with positively charged GUVs and disrupt the electrostatic forces responsible for the aggregation. DDAB was instead expected, due to its positive charge, to interact with negatively charged GUVs and disrupt the aggregates. GUV disaggregation was analyzed after an incubation period of approximately 30 min with varying concentrations of oleic acid, SDS, and DDAB.

To track the behavior of the vesicles throughout the experiment and detect potential fusion events, each of the two initial artificial vesicle populations, bearing opposite surface charges, was labeled with a distinct internal dye (CF405S maleimide and CF555 maleimide).

Figure 3 illustrates the effects of adding various differently charged detergents to GUVs–GUVs aggregates at concentrations ranging from 0.01 mM to 1 mM.

**Figure 3:**
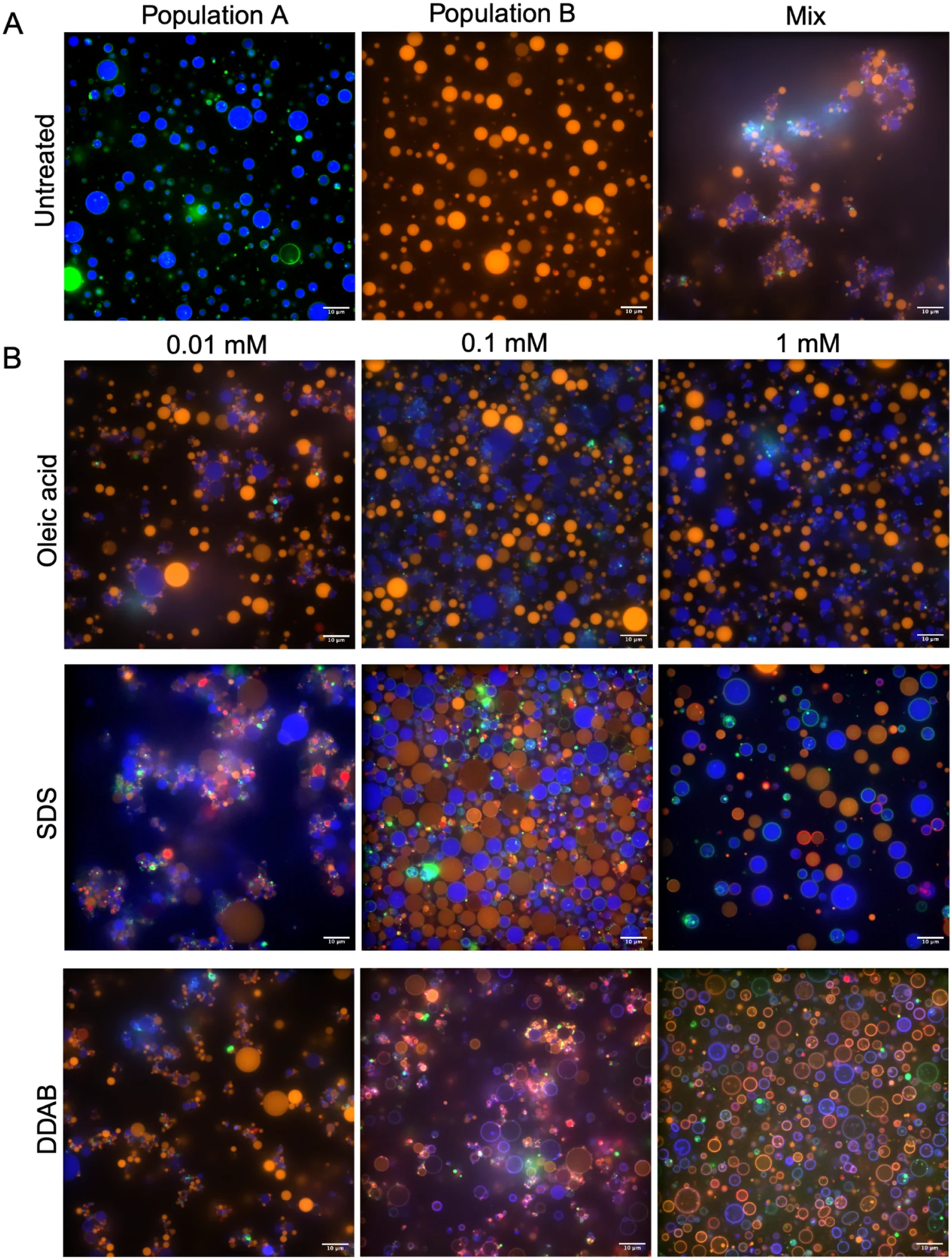
Aggregation reversibility test. (A) Two oppositely charged GUV populations were prepared and mixed. Population A carries a membrane enriched in NBD-PE and POPS with an internal aqueous core containing the dye CF405S-maleimide, whereas population B has a membrane enriched in Cy5-PE and DOTAP with an internal core containing CF555-maleimide. (B) The mixed GUVs were then incubated with ionic species of different charges: oleic acid, SDS, and DDAB. The induced disassembly of GUV aggregates was assessed upon the addition of each species at final concentrations of 0.01, 0.1, and 1 mM. Scale bar: 10 μm.

As shown in the figure, treatment with oleate, SDS, or DDAB led to the disassembly of the preformed aggregates, although to varying degrees. Among the tested detergents, oleic acid was the most gentle: it effectively disassembled the aggregates without significantly reducing the overall number of vesicles in the sample, unlike SDS, which caused a noticeable decrease in vesicle count at 1 mM. Notably, oleic acid was effective even at the lowest concentration tested (0.01 mM). In contrast, DDAB strongly interacted with the negatively charged internal dyes, as indicated by the relocation of the orange and blue dyes from the vesicle lumen to the membrane—an effect most prominent at 1 mM. Due to its mild disaggregation effect and minimal disruption of vesicle integrity, oleic acid was selected for further testing as a disaggregating agent for both GUVs–GUVs and GUVs–EVs charge-mediated aggregates. Additional confirmation of oleate’s interaction with the GUV membranes was provided by zeta potential analysis (Figure S1), which showed that oleic acid treatment shifted the initially positive zeta potential of DOTAP-enriched GUVs to a strongly negative value, consistent with the interaction of negatively charged oleate with the vesicle membrane. The effect on EVs’ charge is instead minimal, reflecting the gentle nature of our method (Figure S1).

The effects of oleic acid addition on GUV–GUV and GUV–EV similarly and oppositely charged mixes were analyzed using confocal microscopy and ImageStream analysis, and the obtained results are shown in Figure 4.

**Figure 4:**
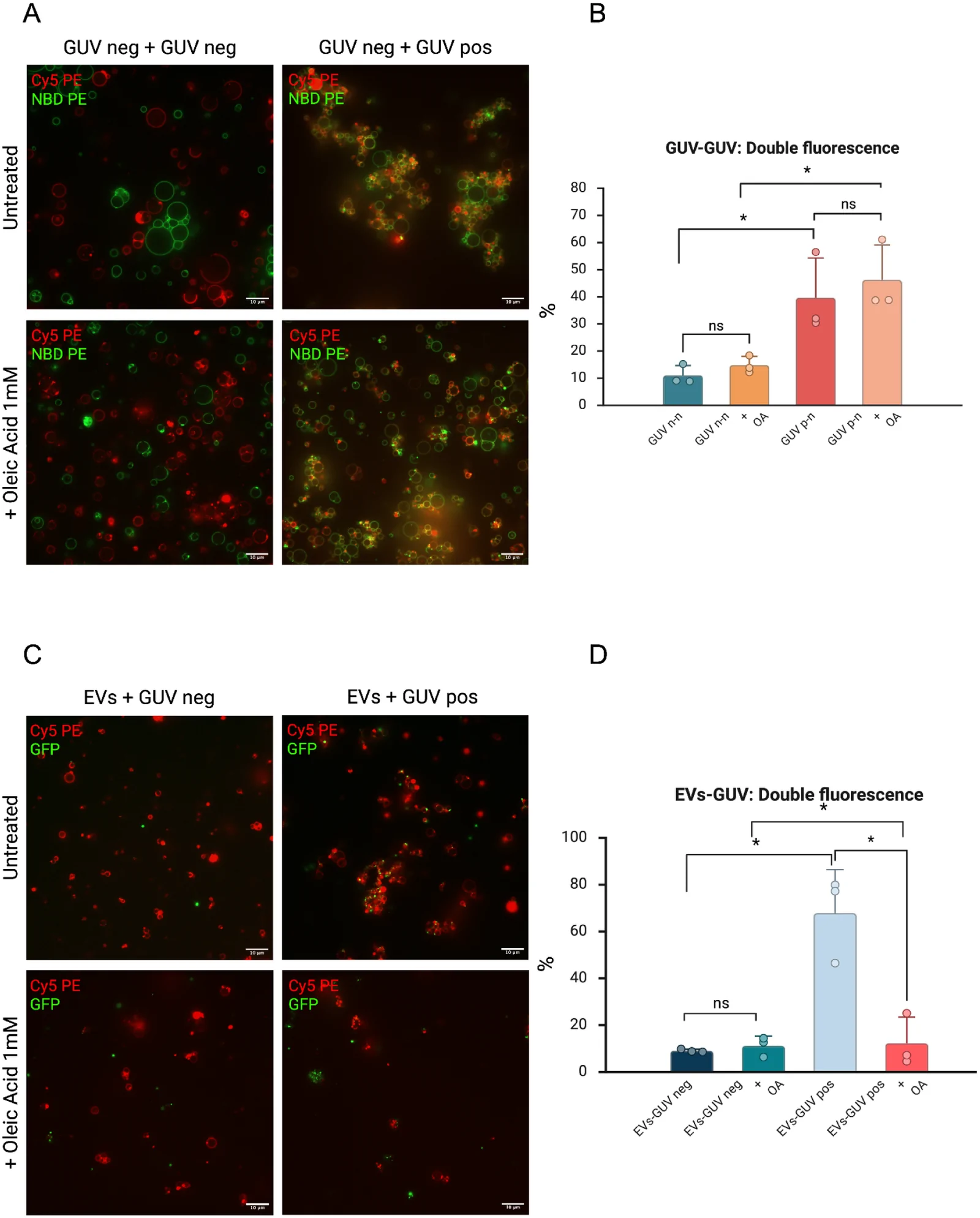
Oleic acid-mediated disaggregation test of GUV–GUV and GUV–EV. Confocal microscopy images showing the effects of oleic acid addition to oppositely or similarly charged GUV mixes (A). (B) Bar graph showing the percentages of objects imaged in (A) with fluorescent signals in both the far-red (Cy5) and green channels (NBD-PE/GFP) in flow cytometry data analysis. Confocal microscopy images of GUVs–EVs mixes before and after oleic acid addition (C) and quantification of the relative flow cytometry data analysis (D). Each experiment was repeated 3 times; error bars represent the standard deviation; *p* > 0.05 ns, *p* < 0.05*, *p* < 0.01**, *p* < 0.001***. Scale bar: 10 μm.

Because during the aggregation reversibility test (Figure 3), no internal dye release was observed after adding oleate, we simplified the system for the subsequent assays using Cy5-PE exclusively to label the GUV membranes, while the EVs contained GFP inside the lumen.

Figure 4 shows the confocal fluorescent imaging and ImageStream disaggregation results obtained on the GUV–GUV and GUV–EV mixes using oleic acid. Confocal imaging revealed that the addition of oleic acid leads to the disaggregation of oppositely charged GUV–GUV and GUV–EV aggregates, while having no effect on similarly charged GUV–GUV and GUV–EV mixes. The sample analysis using ImageStream showed no statistically significant difference in the double-positive fluorescent population between GUV and GUV aggregates before and after oleic acid treatment. This apparent discrepancy with the confocal images, which clearly show aggregate disassembly, is explained by concurrent fusion events occurring during disaggregation: upon oleic acid addition, a fraction of the oppositely charged GUVs fuse, generating single vesicles that carry both membrane dyes. Since ImageStream quantifies the fluorescence of each individual object, a fused vesicle is recorded as a single double-positive event, so the double-positive percentage remains high even though the macroscopic aggregates are no longer present. Confocal microscopy, by contrast, spatially resolves the loss of the aggregated structures and allows the fused single vesicles to be identified directly (Figure 5A).^33–35^ For GUV–EV aggregates, the confocal images show the breakdown of aggregates upon oleic acid addition; the quantitative variation detected by ImageStream is statistically significant and the double fluorescent signal mean is halved, even if the standard variation directly related to the variable final size of the aggregates is high (GUVpos-EVs 67.86 ± 18.55 before oleic acid and 12.29 ± 11.16 after oleic acid). The same experimental protocol was further validated using a second GFP-expressing EV population derived from the H1975 cell line. The variation in double fluorescence before and after oleic acid treatment was evaluated using ImageStream analysis and found to be statistically significant in this case as well (see Figure S3; 50 ± 2% before and 10.9 ± 3.3% after oleic acid treatment). To quantify the separation efficiency, we calculated the following metric based on the variation in the percentage of double-positive events: normalized change in double-positive events, expressed as the percentage decrease relative to the pretreatment value: (DP_before – DP_after)/DP_before × 100. The mean separation efficiency for U87-derived EVs was 80 ± 11%, while for EVs obtained from the H1975 cell line it was 78 ± 7%, demonstrating consistent performance across both cell lines.

**Figure 5:**
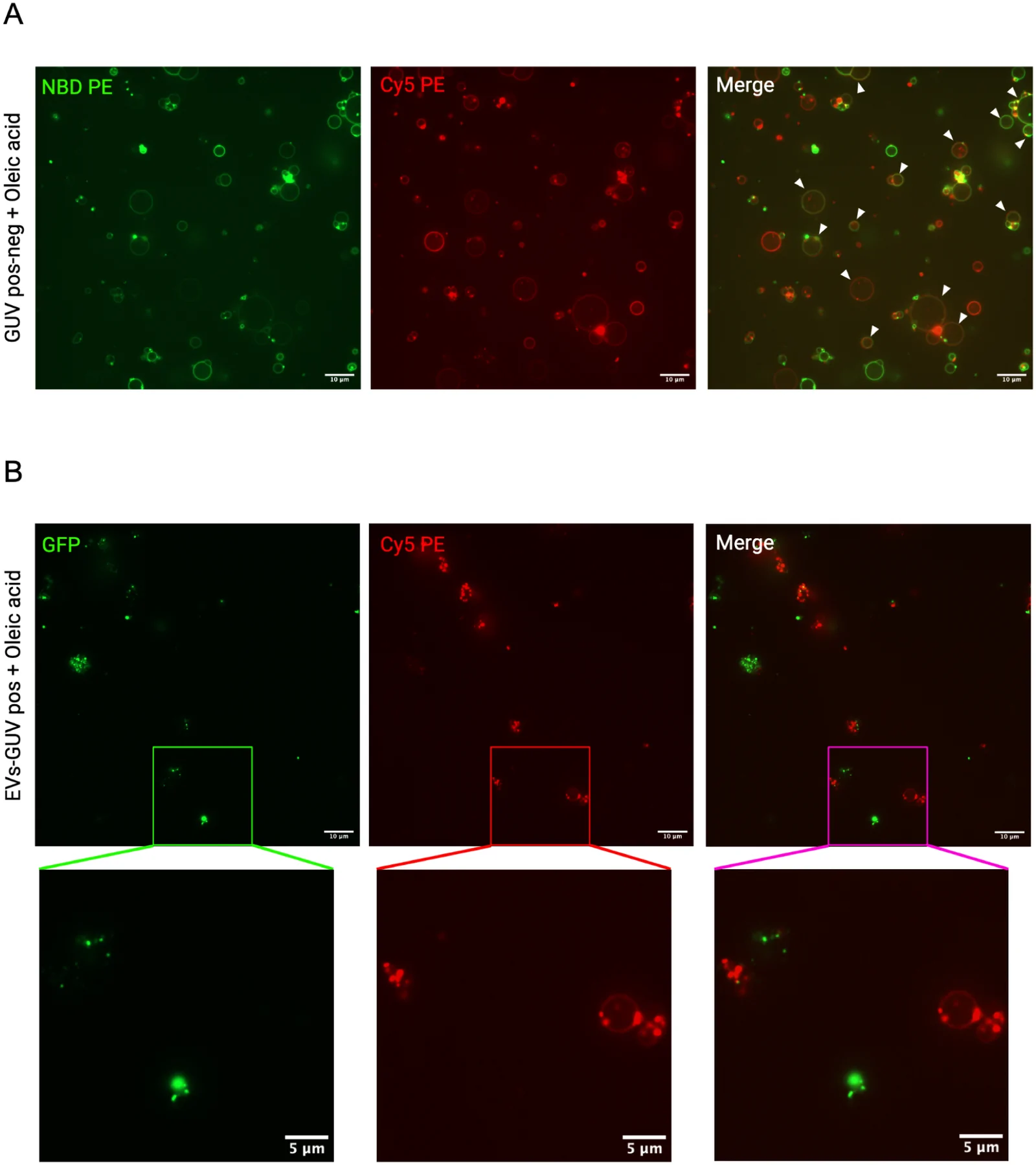
Oleic acid-mediated fusion. (A) Oleic acid-mediated fusion of oppositely charged GUVs; white arrowheads indicate the fused GUVs, showing both green and far-red fluorescence. (B) Absence of fusion when GUVs–EVs mixes are treated with oleic acid. Scale bar: 10 μm.

As previously stated, even if the GUV–GUV aggregates treated with oleic acid were almost completely disassembled (Figure 4A), the flow cytometry analysis of these samples did not prove a statistically significant difference between untreated and treated samples (Figure 4B) due to fusion events. To directly verify the occurrence of fusion during disaggregation, additional microscopy analysis was performed (Figure 5A).

As can be observed in Figure 5, fusion events occurred when GUV aggregates were incubated with oleic acid (Figure 5A), confirming that the persistence of the double-positive signal in the GUV–GUV system reflects fusion rather than residual aggregation. In contrast, no fusion was observed in GUV–EV aggregates treated with oleic acid (Figure 5B). It should be noted, however, that microscopy has a reduced detection limit compared to ImageStream, where a higher double-positive signal is observed. This experiment shows no evidence of compromised EV integrity and identity during the hybrid system formation and disaggregation.

The experiments described in this manuscript demonstrate the feasibility of developing a system that allows reversible aggregation and disaggregation of EVs. These findings suggest that the EVs’ structural integrity is not compromised throughout each step. As a result, they enable precise and controlled manipulation of EVs while maintaining their native properties.

## Discussion and Conclusions

In this study, vesicles with tailored surface charges were developed, enabling controlled interactions based on electrostatic principles. These charged GUVs were shown to selectively form aggregates when mixed with GUVs or EVs of the opposite charge. Specifically, EVs, which are negatively charged, can be effectively captured through electrostatic interactions with positively charged GUVs. Importantly, the interaction was proven to be reversible, as demonstrated by the disaggregation of the complexes upon the introduction of additional negatively charged components, such as oleate.

Other charge-based methods have been used in the past to interact with EVs; however, these approaches are not reversible and rely on interactions with polycationic polymers^36^ or cationic proteins such as protamine.^37^ In all these cases, the positive charge moiety remains bound to the EVs after isolation, making the charge-based interaction irreversible. In contrast, the present study utilizes a reversible charge-based approach, in which only the surface of GUVs is modified through interaction with oleate ions. To contextualize our approach, Table 1 summarizes the key performance metrics of our method alongside representative literature-derived values for established EV isolation techniques. It is important to emphasize that this comparison is intended as a qualitative contextualization rather than a direct, head-to-head experimental evaluation: the capture efficiency, release efficiency, purity, and EV integrity of both conventional methods and our own approach can vary substantially depending on the sample type and protocol.

**Table 1:** Comparative Analysis of Key Performance Metrics across Established EV Isolation Methods and the Charge-Mediated GUV–Based Approach Presented in This Study^a^

Method	Capture Efficiency	Release Efficiency	EV Integrity/Purity	Processing Time	Cost/Equipment	Scalability/Complexity	Key Limitation
Size-Exclusion Chromatography (SEC)	Moderate–high (40–80%)	N/A (flow-through elution)	High purity; gentle on membrane integrity; protein separation	1–3 h	Moderate (columns + FPLC optional)	Limited scalability; EV dilution in eluate	Size-only discrimination; no subpopulation selectivity[ ^15,17^ ]
Immunoaffinity Capture	High (marker-dependent)	Partial (harsh elution needed)	High purity for target population; antibody remains bound after elution	2–12 h (incl. incubation)	Very high (antibody production)	Low throughput; target-specific, not universal	Permanent labeling; alters EV surface; high cost[ ^16,39^ ]
Cationic Polymer Precipitation (e.g., PEI, protamine)	High	None (irreversible)	Low purity; polymer coating permanently modifies EV surface charge	1–2 h	Low (simple reagents)	Scalable; simple protocol	Irreversible binding; polymer alters EV properties; poor purity[ ^36,37^ ]
Anion-Exchange Chromatography (AEX)	High (>70%)	Partial (salt/pH gradient)	Moderate purity; high salt in eluate may affect downstream use	1–4 h	Moderate (column + FPLC)	Scalable; moderate complexity	Harsh elution conditions; EV modification risk; nonselective[ ^22,23,25^ ]
GUV Charge-Mediated Capture (this study)	Moderate high (50–80% double-positive signal by ImageStream)	∼80% of captured EVs released upon oleate addition	No evidence of compromised integrity; no permanent labeling; slight negative charge variation	∼1 h (30 min aggregation +30 min release)	Low (lipids + oleic acid; no specialized equipment for capture/release step)	Moderate (GUV preparation required); adaptable to FACS-based separation	Charge-based interaction low efficiency in media with normal/high ionic strenght; GUV preparation variability

a Capture efficiency reflects the proportion of EVs bound from the initial population; release efficiency (where applicable) reflects the proportion of bound EVs successfully recovered. EV integrity refers to the preservation of native membrane properties and cargo. ND = not determined; N/A = not applicable.

The dynamic control of our system was achieved through the careful selection of charged surfactants, which are essential for reversible interactions. However, not all charged surfactants perform equally well in this context. For instance, while sodium dodecyl sulfate (SDS) effectively induces vesicle disassembly, it also tends to solubilize a portion of the vesicles, compromising their structural integrity. In contrast, oleate ions offer a superior alternative by enabling robust yet reversible surface charge modification without significant vesicle disruption. This selective and gentle interaction ensures both efficient assembly control and the preservation of vesicle functionality, highlighting oleate’s distinct suitability for applications requiring reversible charge-based modulation. It should also be noted that the experiments presented here were performed in a low-ionic-strength solution. In physiological fluids such as plasma or PBS, the higher ionic strength would be expected to weaken both the GUV–EV aggregation and the oleate-mediated release. Consequently, application of this approach to complex biological fluids will require adaptation of the working conditions to preserve the charge-mediated interactions. This salt sensitivity is also reflected in Table 1 as a key limitation of the method.

More complex assembly methods have also been explored in the past, such as DNA-mediated systems^38^ and immunocapture;^39^ these systems can provide higher specificity and, in some cases, have shown partial reversibility.^40^ However, the charge-mediated assembly and oleate addition-based method presented here stand out for their low cost and simplicity, as well as their adaptability to different kinds of platforms.

As a result, charge-mediated interactions between GUVs as well as between GUVs and EVs represent a powerful and versatile strategy to separate oppositely charged aggregates. This approach is easy to engineer, requiring only simple modifications to the membrane composition to control its surface charge. Unlike ligand–receptor binding mechanisms, charge-driven interactions do not depend on highly specific molecular recognition, making them broadly applicable across various systems. Furthermore, these interactions are noncovalent and reversible, allowing for dynamic control over assembly and disassembly. The observed fusion events in GUV–GUV systems highlight the potential to modulate the internal composition of aggregated structures, while the absence of fusion in GUV–EV systems demonstrates the reversibility, with the promise of designing innovative hybrid platforms where GUVs are linked to EVs and in which EVs remain structurally unaltered. This feature could be exploited to allow labeled GUVs to capture unlabeled EVs, enabling their separation via FACS without any evidence of compromised EV integrity. EVs, using this charge-based method, could be released after FACS separation while remaining unaltered, something that is currently not possible with lipophilic dyes such as MemGlow, which irreversibly stain EVs and potentially vary their interaction with cells.^41^

Looking ahead, several directions emerge from our work. A current limitation is that all experiments were carried out in a low-ionic-strength medium; a key near-term step will therefore be to validate the charge-mediated capture and release in physiological and complex biological fluids, such as plasma or cell-conditioned media, where the higher salt concentration is expected to modulate the electrostatic interactions and may require the optimization of the working conditions. Beyond this, a further objective will be to identify conditions that enable the controlled fusion between GUVs and EVs. Such control would allow the creation of specifically cargo-loaded GUVs that can dynamically capture, manipulate, and release EVs. Together, these advances open exciting new possibilities for engineering EV-based delivery systems and developing responsive therapeutic platforms in which EV functionality could be precisely tuned *in situ*.

## Materials and Methods

### Reagents

1,2-Dipalmitoyl-*sn*-glycero-3-phosphocholine (DPPC), 1,2-dioleoyl-*sn*-glycero-3-phosphoethanolamine (DOPE), 1,2-dioleoyl-3-trimethylammonium-propane (DOTAP), 1-palmitoyl-2-oleoyl-*sn*-glycero-3-phospho-l-serine (POPS), 1,2-dioleoyl-*sn*-glycero- 3-phosphoethanolamine-N-(Cyanine 5) (Cy5 PE), and 1,2-dioleoyl-*sn*-glycero-3- phosphoethanolamine-N-(7-nitro-2-1,3-benzoxadiazol-4-yl) (NBD PE) were purchased from Avanti Polar Lipids (Alabaster, AL, USA). Glucose, sucrose, silicone oil, oleic acid, HEPES powder, sodium dodecyl sulfate (SDS), didodecyldimethylammonium bromide (DDAB), and Repel-silane ES were purchased from Sigma-Aldrich (St. Louis, Missouri, USA). Dulbecco’s Modified Eagle Medium (DMEM), phosphate-buffered saline (PBS) pH 7.4, Fetal Bovine Serum (FBS), l-glutamine, penicillin, and streptomycin were purchased from Gibco, Thermo Fisher Scientific (Waltham, Massachusetts, USA). Puromycin was obtained by Sigma-Aldrich.

### GUV Preparation

The Giant Unilamellar Vesicles were prepared using the inverted emulsion method, with slight modification of a previously published protocol.^40^ Briefly, lipids were mixed in a clean glass vial to obtain a final total concentration of 0.8 mM (final volume: 2 mL), with the following ratio: DPPC-DOPE-DOTAP/POPS-Cy5 PE/NBD PE 67.25-22.5-10-0.25. The chloroform was completely evaporated by a N_2_ stream followed by 40 min of incubation in a vacuum desiccator. The desiccated lipid mix was then resuspended in 2 mL of silicone oil and incubated for 20 min at 80 °C to fully solubilize the lipids. In the meantime, the Hosting solution (HS: 500 mM glucose, 10 mM HEPES in Milli-Q H_2_O) and the Internal solution (IS: 500 mM sucrose, 10 mM HEPES in Milli-Q H_2_O) were prepared and prewarmed to 55 °C in a heat block. A defined volume of lipid-in-oil mix (200 μL) was poured on top of the HS and IS (200 and 20 μL, respectively) and incubated at 55 °C overnight. After complete flattening of the oil–water interfaces, an emulsion of water-in-oil was created by scratching the IS-containing tubes over a tube rack several times; this emulsion was poured on top of the HS-containing tubes and the tubes were centrifuged for 1 min at 16,000 × *g* at 40 °C. The supernatant was removed, and the GUV pellet was resuspended in HS.

### Biological EV Preparation

Extracellular vesicles (EVs) were isolated from the glioma cell line U87-MG purchased from ATCC (HTB-14). These cells were cultured in DMEM supplemented with 10% FBS, 2 mM l-glutamine, and 100 U/mL penicillin + 100 μg/mL streptomycin, and grown at 37 °C with 5% CO_2_ in a humidified atmosphere (∼95%). To stably express TurboGFP (excitation/emission max = 482/502 nm), U87-MG cells were transduced with pCMV6-AN-GFP—a shRNA lentiviral vector with an N-terminal tGFP tag (Origene, PS100019), at a concentration of 1 mg/mL in TE buffer, pH 8.0. Cells were selected using 50 μg/mL of puromycin. After the lentiviral transduction, GFP-positive cells were isolated using the cell sorter BD FACS Aria IIIu (Becton Dickinson Co., Franklin Lakes, New Jersey, USA). An analogous protocol was employed to generate GFP-expressing EVs from the NCI-H1975 cell line (H1975).

Twenty-four hours prior to EV isolation, cells were washed with PBS and grown in serum-free DMEM medium. The secretome from a total of 20 × 10^6^ cells was subjected to a first round of centrifugation at 2800 × *g* for 10 min at room temperature to remove cell debris. This was followed by a single round of ultracentrifugation at 100,000 × *g* for 70 min at room temperature using polypropylene tubes (Beckman Coulter, Brea, California, USA) and the ultracentrifuge Optima XPN-100 with rotor SW32 Ti (Beckman Coulter). Following centrifugation, the supernatant was decanted and EV pellets were resuspended in a hosting solution (500 mM glucose, 10 mM HEPES in Milli-Q H_2_O). EVs were profiled for size and concentration using a NanoSight NS300 (Malvern Panalytical, Malvern, Worcestershire, UK).

### Aggregates Preparation and Treatment

The GUVs populations were freshly prepared on the same day as the experiment; the EVs were either used fresh or thawed after being frozen at −80 °C for a few days. The GUVs were diluted 1:10 in HS and mixed 1:1 with EVs, then left at RT for 30 min. Right before imaging, the mixes were diluted 1:20 for confocal microscopy and 1:10 for the ImageStream acquisition.

For treated samples, SDS, DDAB, or oleic acid at the desired final concentrations were added to the aggregates and incubated for 30′ at RT. Before imaging, the samples were diluted as previously mentioned.

### ImageStream Acquisition

An Image Stream Mark II (Cytek Biosciences, Fremont, California) equipped with 405, 488, and 642 nm lasers and 785 nm laser for SSC, bright field, and a 2-camera configuration was used for the experiments. Data were acquired using the ISPIRE software (version 201.1.0.765). For GUVs and EVs analysis, a 60× magnification and maximum (30 μm) core stream diameter were set. Each sample was diluted in a 0.1 μm filtered hosting solution to have a final volume of 40 μL.

The software IDEAS (version 6.4) was used to analyze the obtained data. The gating strategy is illustrated in the Supporting Information (Figure S2). Each experiment was performed on at least three independent biological replicates (separate GUV preparations and EV isolations). Differences were considered statistically significant at *p* < 0.05, and significance levels are indicated in the figure legends as follows: *p* > 0.05 ns, *p* < 0.05*, *p* < 0.01**, *p* < 0.001***.

### Microscopy

The vesicles were observed in a 96-well optical glass-bottom plate. Images were acquired on a Nikon Ti2 microscope equipped with a CREST Optics X-Light V3 spinning disk module. Samples were imaged with a Plan Apochromatic 100× oil (1.45 NA) objective, and the images were recorded by a Zyla 4.2 CMOS camera (Andor Technology, Belfast, UK). The light was provided by a CELESTA solid-state laser array light engine. The software used for acquisition was NIS Elements AR version 5.42.06. Each experiment was performed on three independent biological replicates.

### NTA Analysis

The EVs’ concentration and size distribution were verified using the nanoparticle tracking analysis (NTA) system Nanosight NS300 (supplied by Malvern Panalytical). The instrument is equipped with a 532 nm laser and a syringe pump.

The samples were diluted (1:250) to a final volume of 1 mL with 0.22 μm filtered hosting solution, in order to achieve an optimal particle-per-frame value (20–120 particles/frame). The camera captured and recorded 5 videos of 1 min each, with an intensity value set to 13. The results were processed and visualized with the software NanoSight NTA 3.4. All quantitative data are expressed as mean ± standard deviation (SD). Comparisons between two experimental groups were performed using a two-tailed Student’s *t*-test.

### Zeta Potential Measurement

To measure the zeta potential of the GUVs and EVs, the samples were diluted in the hosting solution to obtain a final volume of 1 mL. This total volume was then used to fill a folded capillary cell (Malvern Panalytical), which was capped and loaded into a Zetasizer Nano ZS (Malvern Panalytical) instrument. The software Zetasizer K (version 7.13) was used to analyze the samples, with the following settings: material (DPPC) refractive index 1.478, absorption 0.001; dispersant (hosting solution) viscosity 1.172, refractive index 1.343, dielectric constant 78.5. Samples were acquired at 25 °C after an equilibration of 60 s. Each experiment was performed on three independent biological replicates.

## Supplementary Material



## Data Availability

All data needed to evaluate the conclusions in the paper are present in the main text and/or the Supporting Information.

## Notes

Figure S1: Zeta potential analysis of positively and negatively charged GUVs and of EVs (U87, H1975), with and without oleic acid treatment; Figure S2: Gating strategy for ImageStream analysis of GUV and EV populations; Figure S3: Percentage of double-positive (Cy5/GFP) events in flow cytometry analysis of GUV–EV mixed populations before and after oleic acid treatment (PDF)
