# Supplementary material for: Reversible Charge-Mediated Capture and Release of Extracellular Vesicles Using Giant Unilamellar Vesicles
Source: ACS Omega. 2026 Aug 13;11(33):49033–44. doi: 10.1021/acsomega.6c00387 (PMC13520013; doi:10.1021/acsomega.6c00387)
Supplement: Supplementary file 1 [file ao6c00387_si_001.pdf]

SUPPLEMENTARY DATA

Reversible Charge-Mediated Capture and Release of Extracellular Vesicles Using Giant Unilamellar Vesicles

Marinella Pinelli[1], Gabriele Schiavi[1], Dalia Tarantino[1], Luca Valer[1], Martina Cortese[1], Michela Notarangelo[1], Miriam Romano[2,3], Annalisa Radeghieri[2,3], Lucia Paolini[3,4], Paolo Bergese[2,3], Vito G. D'Agostino[1], Martin M. Hanczyc[1,5], and Silvia Holler[1]*

[1]Cellular Computational and Integrative Biology Department, CIBIO, University of Trento, Via Sommarive 9, Povo, 38123, Italy

[2]Department of Molecular and Translational Medicine, University of Brescia, 25123 Brescia, Italy

[3]Center for Colloid and Surface Science, (CSGI), Sesto Fiorentino (FI), Italy.

[4]Department of Medical and Surgical Specialties, Radiological Sciences and Public Health, University of Brescia

[5]Chemical and Biological Engineering, University of New Mexico, Albuquerque, New Mexico 87106, United States

A.

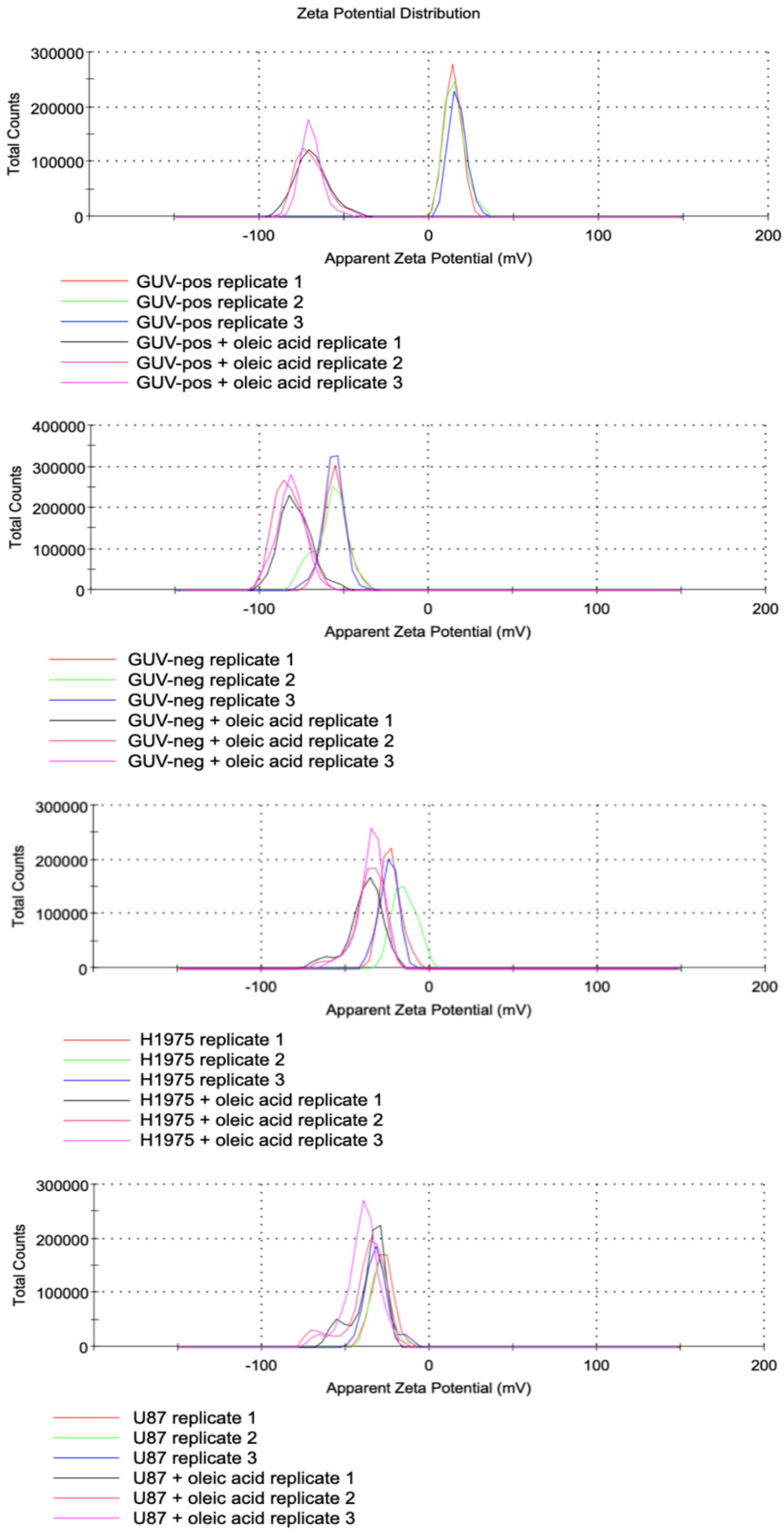
Zeta Potential Distribution
Total Counts
300000
200000
100000
0
-100
0
100
200
Apparent Zeta Potential (mV)
GUV-pos replicate 1
GUV-pos replicate 2
GUV-pos replicate 3
GUV-pos + oleic acid replicate 1
GUV-pos + oleic acid replicate 2
GUV-pos + oleic acid replicate 3
400000
GUV-neg replicate 1
GUV-neg replicate 2
GUV-neg replicate 3
GUV-neg + oleic acid replicate 1
GUV-neg + oleic acid replicate 2
GUV-neg + oleic acid replicate 3
H1975 replicate 1
H1975 replicate 2
H1975 replicate 3
H1975 + oleic acid replicate 1
H1975 + oleic acid replicate 2
H1975 + oleic acid replicate 3
U87 replicate 1
U87 replicate 2
U87 replicate 3
U87 + oleic acid replicate 1
U87 + oleic acid replicate 2
U87 + oleic acid replicate 3

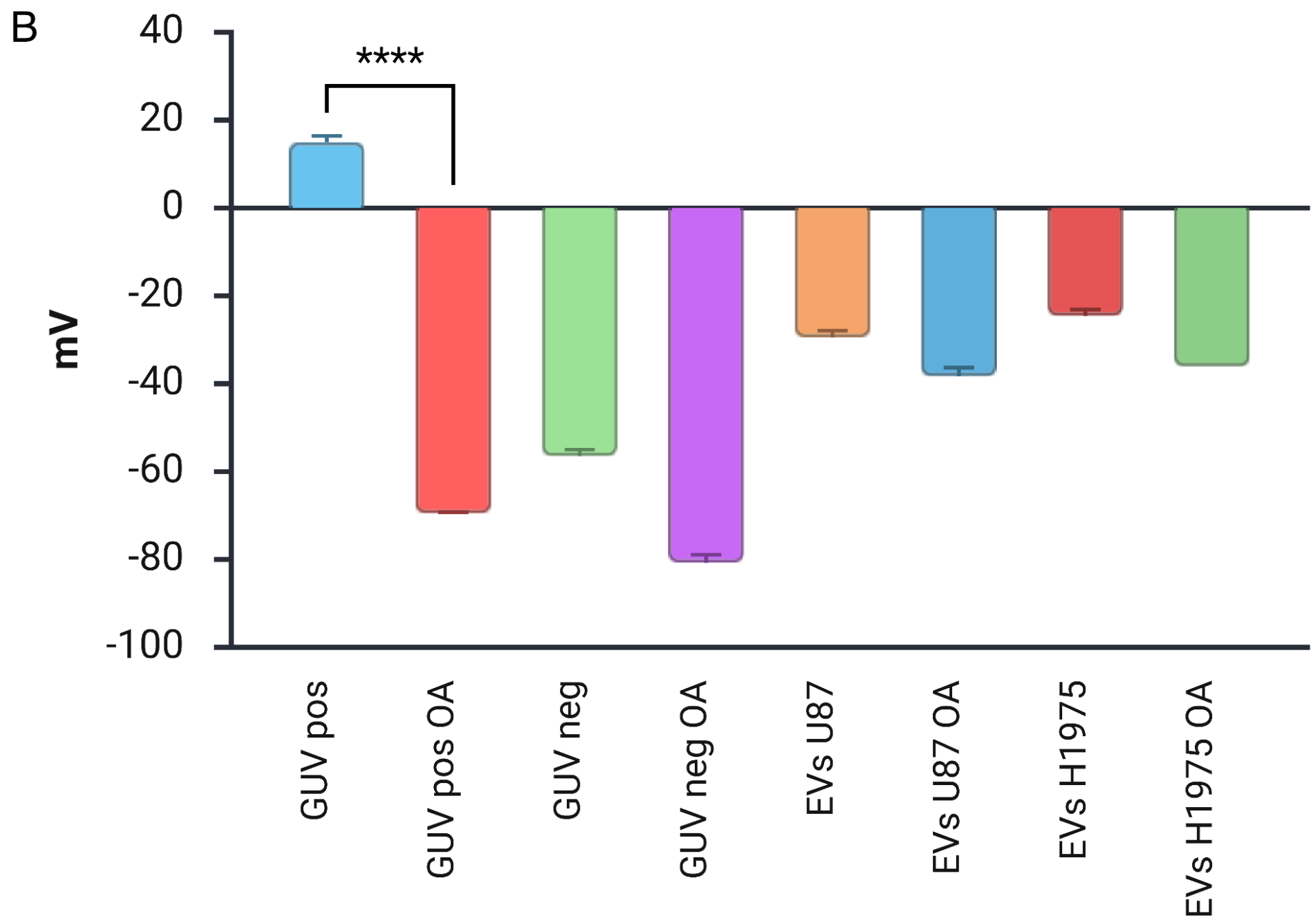

Figure S1: Zeta potential analysis of positively charged GUVs, negative charged GUVs, EVs U87 and EVs H1975 without and with oleic acid treatment. Panel (A) shows the distribution of the zeta potential of 3 replicate measurements of DOTAP-enriched GUVs (mean value 15 ± 1.4 mV), 3 replicate measurements of DOTAP-enriched positive GUVs treated with 1mM oleic acid (mean value -69,2 ± 0.3 mV), POPS-enriched negative GUVs (mean value -56.5 ± 1.5 mV), 3 replicate measurements of POPS-enriched GUVs treated with 1mM oleic acid (mean value -80.9 mV ± 1.9 mV), EVs U87 (mean value 29.5 ± 1.5 mV), 3 replicate measurements of EVs U87 with 1mM oleic acid (mean value -38.3 ± 2 mV), EVs H1975 (mean value -24.5 ± 1.5 mV), EVs H1975 treated with 1mM oleic acid (mean value -31.5 mV ± 0.6). The quantification is represented in the bar graph in panel (B), which clearly shows the significant decrease of the zeta potential in only the sample of GUVs positive after the treatment. The z potential variation following oleic acid treatment was observed for GUV-positive particles was 84.1 ± 1.7%. The variation recorded for GUV-negative particles was 24.4 ± 3.4%, while for U87-derived EVs it was 8.9 ± 3.6%, and 11.5 ± 2.0% for H1975-derived EVs.

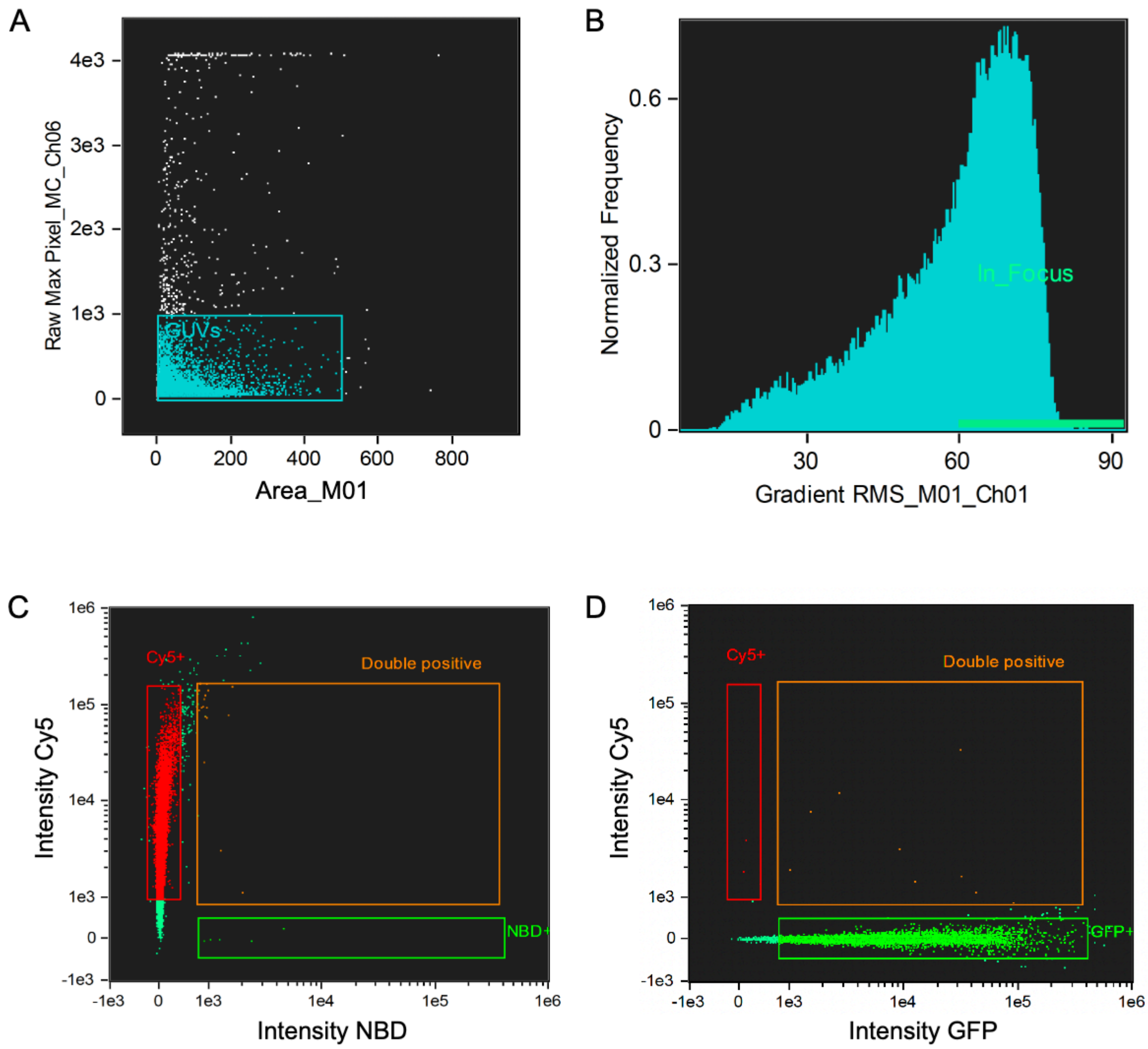

Figure S2: Gating strategy for the Imagestream analysis. In panel (A) a gate was created to exclude the SpeedBeads and select only the vesicles, based on the area on the forward scatter channel (Ch01) and the values of pixel on the side scatter (Ch06). Subsequently, only in-focus events were included by selecting those with a Gradient RMS value greater than 60 (B). The in-focus population was then plotted to select the $Cy5^+$ and GFP/NBD^+ populations. Panel (C) and (D) represent the $Cy5^+$ GUVs and the GFP^+ EVs single population, respectively, which have been used to set the single and double positive gates.

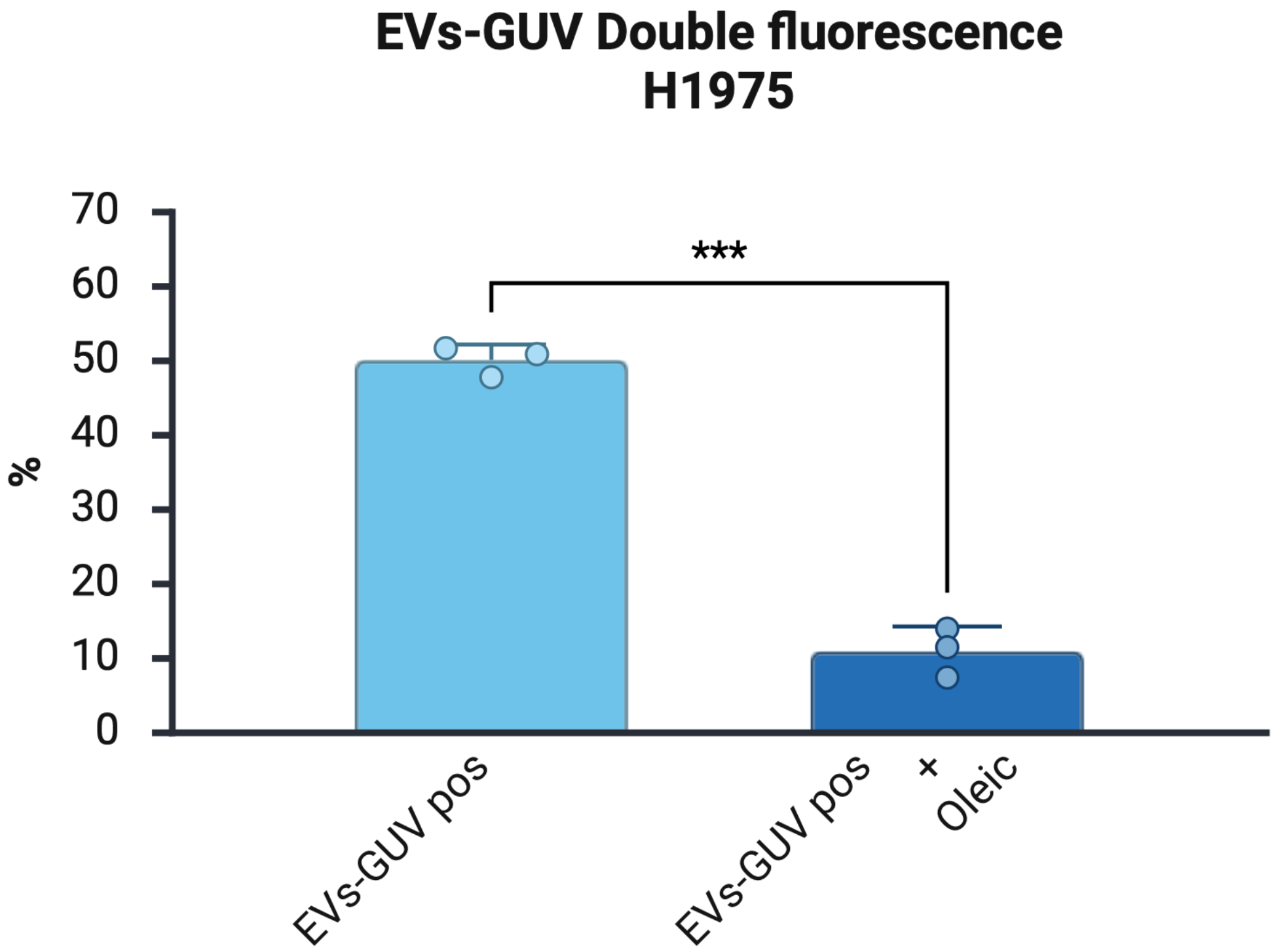

Figure S3: Bar graph showing the percentages of events having fluorescent signal both in far red (Cy5, GUVs) and green channel (GFP, EVs) in flow cytometry data analysis for a mixed population of GUVs positively charged and EVs H1975 before and after oleic acid treatment.
